# Health care utilization among rural women of child-bearing age: a Nigerian experience

**DOI:** 10.11604/pamj.2015.20.151.5845

**Published:** 2015-02-17

**Authors:** Titilayo Dorothy Odetola

**Affiliations:** 1 Department of Nursing, University of Ibadan, Ibadan, Nigeria

**Keywords:** Choice, experience, child-bearing age, health care

## Abstract

**Introduction:**

Despite the availability of primary health care services in virtually every community and village in Nigeria, clients travel long distances to reach secondary and tertiary health care institutions. Against this backdrop, the researcher sought to find the factors that influence choice of health institutions among pregnant women.

**Methods:**

A descriptive study was carried out across three levels of health care institutions with a total sample size of 160 clients who were randomly selected. A thoroughly validated 45- item questionnaire was used to explore clients’ perceptions about what factors influence where they access health care services.

**Results:**

Major findings revealed that level of education, proximity to place of residence, affordability and quality of services rendered, spousal and significant other's influences were active determinants of choice for health institutions among pregnant women in Nigeria.

**Conclusion:**

This study elicited information on why some types of institutions were preferred. The study has implications for community health nurses and policy makers on what should be done to make health institutions appropriately utilized by community in Nigeria.

## Introduction

Skilled antenatal care and birth attendance has been advocated globally as the most crucial intervention to reduce maternal mortality. Poor usage of skilled attendance and maternal primary health care services results in high levels of maternal mortality in the developing countries. The maternal mortality ratio in Nigeria, for example, is estimated at 560 per 100,000 women [1]. Various studies have been done around the world to identify factors that influence the choice of child-bearing women's health care. Some of the identified factors include cost of services [2–6]; socio-demographic and educational level of the client [7–10]; women's level of autonomy in making health care decisions [2, 5]; physical accessibility to health care services [3, 5, 7, 9–11] and the type of health services rendered, disease pattern and healthcare workers attitude [2–4, 7, 10, 12, 13]. Maternal deaths could be prevented if women were able to access and utilize good quality services, especially when complications arise [14]. However, in reality, most women experience serious barriers to accessing services or even if they do reach them, the services themselves are often of insufficient quality or effectiveness. Also, in recent years, sector-wide strategies focused on skilled attendance have aimed to generate demand as well as augment supply.

Access to information about maternal services should be available in the community to help women make choices about who to see and where to go, as well as decide the type of care they require. Information about family planning services can help reduce unwanted pregnancies and their adverse consequences. Access to health care particularly at the critical time of birth, can help ensure that childbirth is a joyful event [15]. Access means that women can reach maternal health care easily and not be deterred by cost or poor treatment by staff. Women have been seen to travel long distances to access quality health care despite a ready availability of primary health care facilities around where they live, work and school. However, lack of transport makes it difficult for pregnant women or women in labour to reach help quickly. Fees charged for health care often put women off having their babies in hospitals or even seeking help when complications arise. Many women also say they prefer to rely on traditional birth attendants because health workers are rude and unsympathetic. In many cases, decisions about seeking care are made by mothers-in-law, husbands or other family members. In the course of rendering professional nursing services at different hospitals that represent the three tiers of health care institution, the researcher observed that most clients attend any health institution irrespective of the level of the health institution, indication for care or their identified health needs. The researcher wished to find out the factors influencing the choice of health care services among women of child-bearing age despite the availability, affordability, accessibility and proximity of health care institutions to the place where they live or work. The study also strove to test three hypotheses: (i) there would be no relationship between choice of health care institution and quality of health services rendered, (ii) there would be no relationship between choice of health care institution and affordability of services and (iii) there would be no relationship between choice of health care institution and the women's level of education.

## Methods

### Study location and design

This study was carried out in Ibadan, Nigeria which has the three categories of health institutions- tertiary, secondary and primary. Ibadan is the capital city of Oyo State and the third largest metropolitan area- by population, with a population of 2,800,000 (NPC, 2006). The principal inhabitants of the city are the Yorubas (FIBSU, 2010). One health facility from each category was randomly selected from the list. The research design was non experimental and focused on identifying the factors that determined the choice of health service institutions among the three selected categories.

### Sampling

The study population included women of child-bearing aged between 18 and 50 years attending the antenatal clinics of the selected health care institutions. A total of 328 women of child-bearing age attend the selected health institutions on a monthly basis. The study was conducted between March and April, 2013 where a representative sample of 160 was randomly selected from the 328 attending the three health centres for an interview.

### Study instrument

A well-structured weighted questionnaire of 38 items involving open- and close-ended questions was used to interview the targeted population group. The first part of the questionnaire focused on the socio-demographic data (8 questions) while the second part focused on the factors influencing choice of health service institution by the child-bearing women (37 questions). The face and content validity of the questionnaire were ensured while a test-retest method was adopted to determine the reliability of the instrument within a two- week interval. The reliability was determined by comparing the two results after administration with a Chrobach's Co-efficient of 0.85.

### Ethical considerations

Ethical approval to conduct the study was obtained from the University of Ibadan/University College Hospital ethical committee and the management of each selected health institution. The participants’ informed consent was sought with an assurance of their confidentiality and anonymity. The questionnaire was administered by the researcher to individual participants of the selected group and retrieved on the spot. Filling in of the questionnaire took between 15 and-20 minutes and the data collection procedure lasted for four weeks.

### Data management

Descriptive statistics such as tables and figures (pie charts and bar charts) were used in representing the identified factors influencing the choice of health care institution while inferential statistics such as correlation analysis was used to test the hypotheses.

## Results

### Socio-demographic characteristics of respondents

The modal age group of the respondents was 21-30 years (52.5%) and most respondents were married (94.4%). Larger percentages (52%) of the respondents were Christians and most of them (37.5%) had a tertiary education. The study revealed that 43.8% earned an income of less than N11, 000 while 22.5% earn over N40, 000. This could be traceable to the fact that 41% were traders. Table 1: from this table, 42.5% of the respondents agreed that proximity influences their choice of health care institution while a larger percentage (65.3%) of respondents claimed that affordability of services delivered played a key role in their choice of health care institution. In the same vein, 77.7% and 84.4% of the respondents believed that their significant others and the quality of services delivered influence their choice of health institutions. However, some of the study population (44.4%) disagreed that their past experiences influenced their choice of attending those health facilities.

**Table 1:** Influence of variables in determining choice of health care institutions

Responses	Frequency	Percentage
**Proximity of health care institution influences choice of health institution**		
Strongly Agree	68	42.5
Agree	35	21.9
Strongly Disagree	27	16.9
Disagree	16	10
Undecided	14	8.8
**Affordability of health care influences choice of health institution**		
Strongly Agree	49	30.6
Agree	55	34.4
Strongly Disagree	29	18.1
Disagree	12	7.5
Undecided	15	9.4
**Spouse and other significant person influence choice of health institution**		
Strongly Agree	91	56.9
Agree	38	23.8
Strongly Disagree	4	2.5
Disagree	9	5.6
Undecided	18	11.3
**Level of education influences choice of health institution**		
Strongly agree	55	34.4
Agree	46	28.8
Strongly Disagree	12	7.5
Disagree	12	7.5
Undecided	35	21.9
**Past health experiences influences choice of health institution**		
Strongly Agree	35	21.9
Agree	14	8.8
Strongly Disagree	26	16.3
Disagree	45	28.1
Undecided	40	25
**Quality of services rendered influences choice of health institution**		
Strongly Agree	76	47.5
Strongly Disagree	4	2.5
Disagree	7	4.4
Undecided	14	8.8
Total	160	100

### Hypotheses testing

Table 2: the above table indicates that there is a relationship between quality of service rendered and the choice of health institution. The negative sign in the correlation coefficient indicates that as one variable increases the other decreases. In essence, an increase in the number of people who choose a particular health institution could bring about a decrease in the quality of service rendered. Table 3: this indicates that there is a relationship between affordability of service rendered and the choice of health institution. The positive sign in the correlation coefficient indicates that as the value of one variable increases, the value of the other variable increases; as one decreases the other decreases. In essence, an increase in the ability to afford services increases the choice of health institutions as a decrease in affordability also decreases choice. Table 4: from the matrix table, the correlation coefficient is 0.197 while at the two tailed; it shows a significance level of 0.013 with a correlation level of 1. This indicates that there is a significant relationship between level of education and child bearing women's choice of health care service.

**Table 2:** Test of significant relationship between quality of health service rendered and choice of health care institution

Variable	N	Mean	Standard deviation	X^2^	P-Value
Choice of health care institution	160	2.1188	0.70374	1	0.002
Quality of health services rendered	160	1.9000	1.20898	-0.245^**^	0.002

** Correlation is significant at the 0.01 level (2-tailed)

**Table 3:** No significant relationship exists between affordability of service and choice of health institution

Variable	N	Mean	Standard deviation	X^2^	P-Value
Choice of health care institution	160	2.1188	0.70374	1	0.001
Affordability of service rendered	160	2.3063	1.24384	0.332^++^	0.001

++ Correlation is significant at the 0.01 level (2-tailed)

**Table 4:** No significant relationship exists between childbearing women's choice of health care service and their level of education

Variable	N	Mean	Standard deviation	X^2^	P-Value
Choice of health care institution	160	2.1188	0.70374	1	0.013
Educational level	160	2.5375	1.55360	-0.197^+^	0.013

+ Correlation is significant at the 0.05 level (2-tailed).

## Discussion

Proximity as a very strong determinant of choice of health institution to the child bearing woman cannot be over emphasized; this implies that hospitals especiallyprimary health centers, when sited 5km from place of residence/work, is likely to receive high turnout of clients. According to Babar and Juanita, 2007, the effect of distance on service use becomes stronger when combined with the dearth of transportation and with poor roads which contribute towards increase costs of visits. And Line et al, 2007 supported the impact of proximity by saying ‘availability of the transport, physical distance of the facility and time taken to reach the facility undoubtedly influences the health seeking behavior and health services utilization’. Wakama, 2003; Al-Nahedh, 2009; Mpembeni et al, 2007 and Rasha and Mansoura, 2007 from the empirical studies indicated in their different research work that location of the hospital and distance a determinant of choice of health care service institution. The result of this study also showed that when services are rendered within the economic power of the consumers of health services, people will be able to access the services adequately. The result obtained supports the previous research studies carried out on the above subject, Jumbo, 2002 in her study listed cost of service as a factor influencing women's choice. Likewise Wakama, 2003 listed affordability as a strong determinant while Rasha and Mansoura, 2007 saw socio-economic status as paramount to the choice. Going by the findings of this study, the influence of respondents’ spouses on their choice cannot be under estimated. The result of the study revealed that 77.7% of the respondents reiterated their spouses’ influence had a great impact in their choice of the hospital they attend. This means that support from significant others especially the spouse plays a great significant role in the respondents’ seeking care in a particular health care institution not taking in consideration the level/tier of the institution. This stance was also supported by Babar and Juanita, 2007 wherein most women were seen as being socially dependent on men; this might be due to lack of economic control to reinforce her dependency which generates a ripple effect on decision making authority in household chores and choice of health care services.

The result of this study indicated that majority of respondents (63.2%) have their choice of health institution influenced by their level of education. This is quite true as peoples’ decisions most of the time are influenced by their level of education as highly educated childbearing women for example will patronize the best health institution based on their informed minds and perhaps affordability of the service. This is also because the highly educated respondents are likely to earn more to afford even the service cost of the institutions of their choice. Past research work reviewed in this study agreed with the result above as Rasha and Mansoura, 2007 revealed in his work that education is one of the strongest determinants of choice of health care institution. Tarex, et al, 2007 found out that women with higher autonomy and higher level of education are more likely to use health care services than others. From the study, past health experiences of respondents was found not to necessarily influence their utilization of health care facilities. Though, in the course of the study, it was found that some people who regularly attend the primary level of health care seek care in the secondary or tertiary health care institution either as a result of complications during their previous pregnancy or delivery; however, some women chose higher institutions due to services rendered and other factors not necessarily because they had problems in their past pregnancies or deliveries. None of the researcher's reviewed literature discusses this. The importance of the quality of service rendered in any health institution cannot be over emphasized. From the study, most clients (84.4%) make their choice of health care institution based on the quality of services rendered by such institutions. The services are in terms of material resources, obstetric emergency, laboratory services, ambulance services in host of others as well as the availability and friendly attitudes of health care professionals. As earlier indicated, most women of child-bearing age use the secondary and the tertiary health institutions while the primary health institution is being neglected. Despite the cost of services in the secondary and tertiary health institution, they are still being patronized more than the primary health institution. The reason for this disparity is that many of the above named services are lacking in most of the primary health centers. This finding is in agreement with some of the reviewed literatures as Akute 2003, discussed qualified personnel and quality of care as very important factor in the choice of health care institution. Likewise D'Ambruoso et al, 2007 perceived quality of care as a strong determinant of choice of health care institution.

### Implications of findings for midwifery/nursing practice

It is evident from the results of this study that the respondents’ choice of health care institution not influenced by one single factor but combination of factors as most of the factors considered were found to significantly determine their choices. This confirms the ecological approach to care. Empowerment of women is critical to choice of health services and securing safe motherhood because it enables women to: Articulate their health needs and concerns; Access services with confidence and without delay; Seek accountability from service providers programme managers, and from the governments for their policies; and Act to reduce gender bias in families, communities and markets. Empowering women means; enabling them to overcome social, economic and cultural factors that limit their ability to make informed choices, particularly in the areas affecting the most aspect of their lives ‘ their reproductive health (Lucas and Gilles, 2003). Women can be taught mother craft and be encouraged to be involved in business to help them economically even if they are civil servants. The midwives/nurses must rise up to this challenge by educating women of child- bearing age at every available opportunity on what the three tiers of health institutions stand for. The midwife/nurse can act as advocate between the policy makers and our women for provision of good road networking, adequate facilities, and skilled attendants etc in the primary health institution to discourage overcrowding of the secondary and tertiary health care institution.

## Conclusion

The utmost goal of every one seeking health care service is to get the best that will put them in a state of optimal health. Health is defined as a state of complete physical, mental and social wellbeing of an individual and not merely absence of disease or infirmities (WHO, 2004). The childbearing woman is not left out in this desire hence, the choice of health institution they perceive as favourable to them is important not minding the proximity of the undesired health care facility. Contributing to body of knowledge is the fact that every nurse should maintain positive therapeutic interpersonal relationship with their clients. The clients need quality service and attitudes of the staff toward them are seen by most as an important factor in influencing their choice. A smile will go a long way to impact positively as non verbal communication is an important aspect the nurse/midwife should never neglect. More so, government can intervene by making training of personnel a standardized one to make sure that all staff are competent to carry out effectively the task that may be required of them and there is need for more greater deployment of health workers with midwifery skills in poor and rural areas not just the big cities (Safe Motherhood, 2008). Provision of adequate functioning medical equipment to the primary health care centres will be a motivating factor for childbearing women's use.

**Recommendations/suggestions for futher studies:** from the research findings, I will like to recommend the followings: health care workers especially midwives and nurses should accord each client unique care as required; the government should ensure adequate funding of the primary health centers for provision of necessary facilities and make health accessible to everyone at the grass root level; the government should create a forum for women of child- bearing age who are in dearth need of the services of the tertiary health institutions following complications at subsidized rate especially when they cannot afford it. This will help to reduce maternal mortality resulting from affordability of services. Further studies can be conducted across the geographical zone of the country to determine the ratio of the people using the three tiers of health care institutions and possible and lasting solution find to people clouding the tertiary health institution that is meant to be a referral and research centre. The problems of distance and lack of transport can be overcome by assigning health workers trained in midwifery to village health posts, by upgrading local health facilities and by organizing emergency transport systems. The problem of cost can be tackled by providing maternal and infant health services free of charge or by adjusting fees to make essential services affordable (Safe Motherhood, 2008). Health workers should be encouraged to show more empathy for women. To facilitate this, health workers need to be shown and offered appropriate support by the health system. Secondary and tertiary health care institutions in Nigeria are often overwhelmed by women seeking services which can be handled at Primary Health Care facilities resulting in overstretched resources and services at the higher health care institutions.
